# Grafted Lactic Acid Oligomers on Lignocellulosic Filler towards Biocomposites

**DOI:** 10.3390/ma15010314

**Published:** 2022-01-02

**Authors:** Anna Czajka, Radosław Bulski, Anna Iuliano, Andrzej Plichta, Kamila Mizera, Joanna Ryszkowska

**Affiliations:** 1 Faculty of Materials Science and Engineering, Warsaw University of Technology, Wołoska 141, 02-507 Warsaw, Poland; radoslaw.bulski.stud@pw.edu.pl (R.B.); joanna.ryszkowska@pw.edu.pl (J.R.); 2 Faculty of Chemistry, Warsaw University of Technology, Noakowskiego 3, 00-664 Warsaw, Poland; anna.iuliano@pw.edu.pl (A.I.); andrzej.plichta@pw.edu.pl (A.P.); 3 Central Institute for Labour Protection—National Research Institute, Czerniakowska 16, 00-701 Warsaw, Poland; kamiz@ciop.pl

**Keywords:** lignocellulosic material, chemical modification, poly(lactic acid) composites, in situ polymerization, grafting biocomposites, biodegradable composites

## Abstract

Lactic acid oligomers (OLAs) were in situ synthesized from lactic acid (LAc) and grafted onto chokeberry pomace (CP) particleboards by direct condensation. Biocomposites of poly (lactic acid) (PLA) and modified/unmodified CP particles containing different size fractions were obtained using a mini-extruder. To confirm the results of the grafting process, the FTIR spectra of filler particles were obtained. Performing ^1^HNMR spectroscopy allowed us to determine the chemical structure of synthesized OLAs. The thermal degradation of modified CP and biocomposites were studied using TGA, and the thermal characteristics of biocomposites were investigated using DSC. In order to analyse the adhesion between filler particles and PLA in biocomposites, SEM images of brittle fracture surfaces were registered. The mechanical properties of biocomposites were studied using a tensile testing machine. FTIR and ^1^HNMR analysis confirmed the successful grafting process of OLAs. The modified filler particles exhibited a better connection with hydrophobic PLA matrix alongside improved mechanical properties than the biocomposites with unmodified filler particles. Moreover, a DSC analysis of the biocomposites with modified CP showed a reduction in glass temperature on average by 9 °C compared to neat PLA. It confirms the plasticizing effect of grafted and ungrafted OLAs. The results are promising, and can contribute to increasing the use of agri-food lignocellulosic residue in manufacturing biodegradable packaging.

## 1. Introduction

These days, environmental issues are becoming key when designing plastic materials. Following the EU Strategy for Plastics in the Circular Economy [1], the goal is to achieve the sustainable management of the product at every stage of its life (extraction, manufacture, use, and disposal). After use, the material should be reused in accordance with the circular economy where nothing is wasted.

The reuse of agri-food lignocellulosic residue fits with this idea. Lignocellulosic residue from the agri-food industry is used as an animal feed to produce biogas or extracts. An interesting form of managing this waste is its use as a natural filler in polymer composites. Such a strategy was proposed by R. Turco et al. [2], where they developed a composite based on poly (lactic acid) (PLA) reinforced by epoxidized oil and presscake waste fibers from oil extraction of the *Cynara cardunculus* plant. Rocha et al. compatibilized natural lignocellulosic residues such as sugarcane bagasse, maçaranduba, and pinus through starch also as a PLA fillers [3]. Mysiukiewicz and Barczewski also used linseed cake to make green composites on the PLA matrix [4]. Many authors have reported using lignocellulosic fibers (not only of waste origin) as fillers for plastics. The most interesting and advantageous in terms of ecological aspects is its use as a filler for biodegradable plastic, such as PLA [5], polyalkanolates (PHA) [6,7]. According to Mohanty et al., a biocomposite created with a biodegradable polymer matrix with a biodegradable filler should allow the obtainment of a biodegradable composite [8]. Using lignocellulosic fillers is also common for fossil plastic, such as polypropylene (PP) [9] and polyethylene (PE) [10,11].

The most promising polymer to replace petroleum-based plastics is PLA, which is a biodegradable aliphatic semi-crystalline polyester obtained in the industry by the ring-opening polymerization (ROP) of lactide (LA) [12]. LA is formed by the dimerization-cyclisation of lactic acid oligomers, which are produced by the condensation of lactic acid (LAc). The latter is usually obtained by the fermentation of agricultural raw materials (saccharides). Due to the non-petroleum origin of LAc, LA, and PLA, and polymer’s ability to biodegradation [13], it is considered as a “double green” polymer. PLA and LA copolymers (e.g., block copolymers [14]) may be used for biomedical or pharmaceutical [15,16,17,18] applications. However, the main market of these polymers is packaging [19,20].

The main purpose of adding lignocellulosic filler in particleboards is to cut down on material cost. However, reinforcement by lignocellulosic fibers such as jute [21,22] or flax [23] may improve the mechanical properties compared to the unfilled polymer matrix. The most basic ingredients of lignocellulose filler are cellulose, hemicellulose, lignin, waxes, pectin, and water-soluble ingredients. The content of these components may vary depending on the growing conditions and the methods determining their content [24].

Cellulose is considered to be the main component of the fiber backbone. It is a polysaccharide with a semi-crystalline structure of D-glucopyranose units. It provides strength, rigidity, and structural stability of the fibers. Hemicellulose is an amorphous, highly branched polysaccharide, associated with cellulose possibly by hydrogen bonds. It is mainly made of hexoses and pentoses of varied chemical structures. The highly polar and hydrophilic nature of cellulose and hemicellulose is due to the presence of a large amount of the hydroxyl group. Lignin, as well as hemicellulose, is an amorphous polymer composed of phenylpropane units [24,25,26]. Thanks to the high content of aromatic rings, it shows a higher hydrophobic character. Additionally, its location between cellulose and hemicellulose chains protect against environmental conditions such as temperature and humidity [27].

Unfortunately, lignocellulosic fillers have a number of disadvantages due to their chemical structure. Due to the presence of numerous OH groups, these fillers are characterized by high water absorption. The highly hydrophilic surface has poor adhesion to the hydrophobic polymer matrix in the polymer composite. This causes the deterioration of the mechanical properties, changes in the dimensions of the composite, and water absorption. One of the methods of improving these properties is the chemical modification of lignocellulosic fillers. Its main purpose is to activate the OH group or introduce new groups. As a result, the surface of the filler becomes more hydrophobic, which increases adhesion to the polymer matrix and even meshes with it [24].

Researchers use various chemical treatments. For example, the alkaline processing of roselle and sugar palm reinforced thermoplastic polyurethane showed that fiber surface modification improved hybrid composites’ mechanical, physical, and thermal properties [28]. A.K. Bledzki et al. [29] proved that the acetylation of flax fiber can reduce water absorption by 42%. The esterification of cellulose with citric acid by X. Cui et al. [30] showed enhanced flexural modulus and stress.

In 2012 alone, 469,200 tons of waste was generated in Poland during the processing and preservation of fruit and vegetables [31]. According to *Statistics Poland* data, more than 50,000 tons of chokeberry were produced in Poland in 2018 [32]. A crucial part of becoming more eco-friendly is reducing the carbon footprint associated with transporting wastes. The reuse of agri-food waste is also significant in protecting the environment. In order to reduce the transport and management costs for locally produced waste, chokeberry pomace (CP) was chosen as a filler.

In this paper, biocomposites based on PLA and modified lignocellulosic filler in the form of CP, which are residue from the food industry, were prepared. The purpose of the modification is to increase its hydrophobicity and the compatibility of the filler with the polymer matrix. The modification consisted of the reaction of free hydroxyl groups present in lignin, cellulose, and hemicellulose with carboxyl groups (esterification reaction) of LAc and its oligomers (OLAs) by the direct condensation of LAc on CP fibers. Additionally, free OLAs formed in condensation polymerization (ungrafted to the lignocellulosic backbone) could act as a PLA plasticizer. Furthermore, the influence of the particle size of lignocellulosic fillers on thermal and mechanical properties was investigated. The main reason was to check whether the change in the proportion of modified filler fraction after modification and grinding affects the properties. It is worth emphasizing that the modification process was made without using organic solvents, which increases the eco-friendliness of the process. This type of modification (by esterification), known as *grafting*, has been the subject of J. Ambrosio-Martín et al.’swork [33] who modified bacterial cellulose nanowhiskers (bacterial CNW). R. Patwa et al. [34] applied a similar procedure for bacterial cellulose modification. Using OLAs as a plasticizer was reported by scientists [35,36,37]. N. Burgos et al. showed that OLAs with a molar mass around 1000 g/mol can be used as a biodegradable plasticizer for PLA, replacing conventional plasticizers [36]. The use of a PLA plasticizer seems necessary due to the high glass transition temperature (T_g_ = 50–60 °C), which causes brittleness and stiffness at room temperature [38].

## 2. Materials and Methods

### 2.1. Materials

Chokeberry pomace (CP) was kindly supplied by the company AGROPOL Sp. z o.o., Góra Kalwaria, Poland. DL-LAc (racemic mixture) was supplied as an 85 wt% aqueous solution by Sigma Aldrich (St. Louis, MO, USA). SnCl_2_·2H_2_O by Sigma Aldrich (St. Louis, MO, USA) was used as a catalyst. DMSO-d_6_ (0.03% TMS, min. 99.8% deuterization degree) was used as a solvent for ^1^HNMR analysis. Chloroform stabilized with amylene, supplied by Pol-Aura (Olsztyn, Poland) was used for Soxhlet extraction. Poly (lactic acid) (PLA) (Ingeo^TM^ 2003D; M_n_ = 108 kg/mol; 4 wt% D-isomer) from NatureWorks LLC (Minnetonka, MN, USA) was used as polymeric matrix. All chemicals were used as supplied without any purification.

### 2.2. Preparation of Chokeberry Pomace Filler

Pre-dried CP (40 °C, 10 days) was milled in sieve mill MUKF-10 (Młynpol P.P.H., Wyszków, Poland) using a sieve with a mesh size of 200 µm. Milled CP was subjected to modification reaction. Before introducing unmodified CP filler into the polymer matrix, it was fractionated using a sieve machine Haver EML 200 Premium Remote (Haver & Boecker OHG, Oelde, Germany) using a sieve with mesh size: 250, 125, and 63 µm. The fraction proportion in the milled CP filler is shown in Table 1. In prepared biocomposites in further analysis, this fraction proportions was **called “Mix”**.

For thermal and structural comparison to modified CP filler, 1.5 g of CP sample was purified using 220 mL of chloroform in Soxhlet extraction for 10 h and dried in 60 °C for 8 h. This step provided a fat-free sample, the same as after purification of modified CP filler (CP-pure).

### 2.3. Grafting of Chokeberry Pomace Using LAc and OLAs

**Figure 1:**
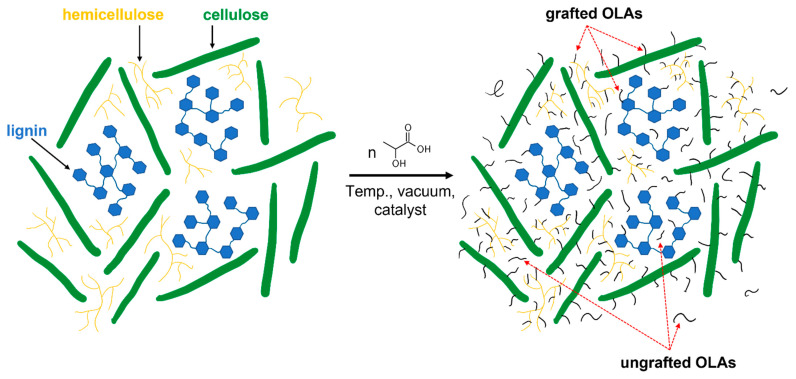
Scheme of chemical modification.

The modification was carried out in a 1:1 ratio (*w/w*) (CP filler: DL-LAc) and 90 ppm of catalyst (for the sum of the reactants). The water content of the CP filler was carried out using a moisture analyzer Axis ATS147-2 from Axis sp. z o. o. (Gdańsk, Poland) (program details: 105 °C; ending parameters: at least 3 measurements with a constant weight every 5 s). The amount of used chemicals is shown in Table 2. All chemicals were added to the two-liter glass reactor. After 2 h of mixing (with constant stirring) under reflux, the solvent was distilled both in atmospheric and under reduced pressure. Then, the condensation process was carried out for 8 h at 160 °C within the pressure range 0.11 to 0.02 mbar. The scheme of the reaction process is shown in Figure 1.

After the modification, the modified filler agglomerated into hard bulks that required shredding. Due to this, bulks of the modified CP filler (CP-g-OLA) were pre-crushed using a hammer and crushed into the powder using a mortal grinder Pulverisette 2 from Fritsch GmbH (Idar-Oberstein, Germany). Grinded CP-g-OLA was fractioned in the same way as unmodified CP filler (Section 2.2). The fraction proportions in the milled CP-g-OLA filler are shown in Table 3. In prepared biocomposites in further analysis, this fraction proportions was **called “Mix 1”**.

To determine the ungrafted OLAs content in the CP-g-OLA and purify the CP-g-OLA for further analysis (CP-g-OLA-pure), the sample was purified in the same way as CP-pure (Section 2.2).

### 2.4. Preparation of Biocomposites

Biocomposites were made using a mini-extruder Haake MiniLab II (Thermo Fisher Scientific, Waltham, MA, USA) equipped with recycle channel and co-rotating conical system of two screws. The mix of filler and PLA granules (5.5 g) were added to the mini-extruder during 3 min of loading time, and it was then mixed for 20 min at a screw speed of 25 rpm in cycle mode in 170 °C. Then, a cylindrical profile formed using Ø 1 mm die was collected for further analysis.

Types of prepared composites are shown in Table 4.

**Table 4:** Manufactured biocomposites composition.

Sample Name	Type of Composite	Filler Quantity, wt%	PLA Quantity, wt%	Ungrafted OLAs Quantity, wt%	Fraction of the Filler, µm
PLA	Neat PLA	0	100	0	-
<63 CP/PLA	CP/PLA	30	70	0	<63
63–125 CP/PLA					63–125
Mix CP/PLA					Mix
Mix1 CP/PLA					Mix 1
<63 CP-g-OLA/PLA	CP-g-OLA/PLA		67.09	2.91 ^2^	<63
63–125 CP-g-OLA/PLA					63–125
Mix CP-g-OLA/PLA					Mix
Mix1 CP-g-OLA/PLA					Mix 1

^2^ Quantity of ungrafted OLAs, LAc, and LA where the main component is OLAs (for more details look at Table 5).

**Table 5:** ^1^HNMR results.

Sample	$\bar{DP}$ _OLA_	M_n_, g/mol	[COOH]/[OH]	χ_LA_	χ_LAc_	χ_OLA_
CP-g-OLA	1.9	281	0.62	0.11	0.09	0.80

It is worth noticing that CP-g-OLA was not purified before the formation of the biocomposites due to the use of ungrafted OLAs containing minor quantities of LAc and LA. The amount of ungrafted OLAs in the biocomposite composition is shown in Table 4, determined in Soxhlet extraction. Also, the CP filler was not purified before the formation of the biocomposites.

### 2.5. Characterisation of Fillers and Biocomposites

The surface morphology of the CP fillers and brittle fracture surface of biocomposites was determined using Hitachi TM3000 SEM (Hitachi Group, Tokyo, Japan). The applied accelerating voltage was 15 kV. All samples were coated using a Polaron SC7640 sputter coater (Quorum Technologies Ltd., Laughton, UK) for 80 s at 10 mA and 1.5 kV with gold and palladium before SEM imaging.

The chemical structure of the CP and CP-g-OLA-pure fillers was studied with the Fourier transform infrared spectroscopy (FTIR) using a Nicolet 6700 spectrometer (Thermo Electrone Corporation, Waltham, MA, USA). Spectral data were collected as a sum of 64 scans in the 4000–400 cm^−1^ range with manual baseline correction and CO_2_ correction. The results were analyzed using the OMNIC 8.2.0 software by ThermoFisher Scientific Inc.

The chemical structure of OLAs was performed by proton nuclear magnetic resonance spectroscopy (^1^HNMR) using the equipment: Varian NMR System 500 (Varian, Inc., Palo Alto, CA, USA). The applied frequency was 500 MHz, measured at room temperature. Before measurement, around 80 mg of CP-g-OLA was diluted in 1.5 mL of DMSO-d_6_ and filtered.

Thermogravimetric analysis (TGA) of the CP-pure, CP-g-OLA, CP-g-OLA-pure, and biocomposites was performed using TGA Q500 (TA Instruments, New Castle, PA, USA). The sample weight was around 10 mg. The sample was heated in ramp procedure (10 °C/min) from room temperature to 650 °C in a nitrogen atmosphere. Data analysis was performed using the Universal Analysis 2000 software, version 4.7 A, by TA Instruments. The measurements were performed using 3 samples.

The scanning of differential calorimetry (DSC) of the biocomposites was performed using DSC Q1000 (TA Instruments, New Castle, PA, USA). A sample weight of 6 ± 0.2 mg was sealed in an aluminum pan. A sample was heated from room temperature to 190 °C with a heating speed of 10 °C/min (first heating cycle); cooling to −80 °C at 5 °C/min (first cooling cycle) and heating to 190 °C at 10 °C/min (second heating cycle) in an inert atmosphere. For further characterization, a second heating cycle was used. The Universal Analysis 2000 software version 4.7 A by TA Instruments was used to determine thermal parameters. The measurements were performed using 3 samples.

The water absorption of modified and unmodified CP fillers was defined as the weight change using TGA. To dry out the fillers, a sample of around 10 mg was heated to 60 °C and held for 180 min. After 24 h, the procedure was repeated.

The mechanical properties of the biocomposites were carried out using Instron 5566 (Instron Norwood, MA, USA) (load cell of 1 kN) tensile testing machine. Cylindrical profiles obtained as a result of extruding were cut into 80 mm long samples and weighed to determine the tex factor. The speed of the tensile strength test was 20 mm/min. The measuring base was 20 mm. The cross-section was determined as a linear mass (tex). To change the unit to MPa, the result in N/tex was multiplied by 900. The measurements were performed using at least 15 samples.

## 3. Results

### 3.1. Analysis of the Chokeberry Pomace Fillers

#### 3.1.1. Chemical Structure of the Fillers

**Figure 2:**
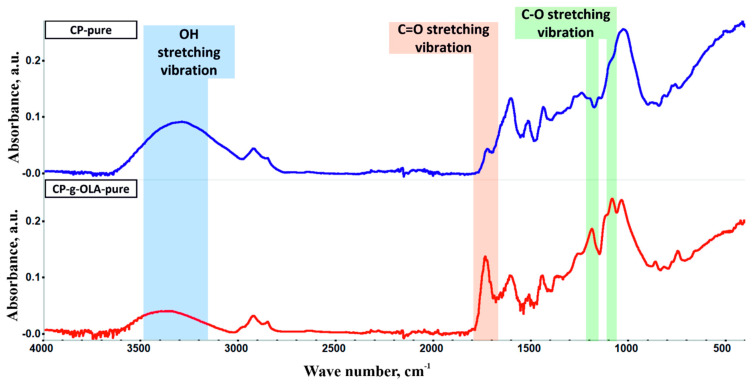
FTIR spectra of CP-pure and CP-g-OLA-pure.

FTIR measurements characterized both CP-pure and CP-g-OLA-pure (after elimination of ungrafted residue) fillers to confirm the chemical reaction between the LAc and hydroxyl groups of lignin, cellulose, and hemicellulose. FTIR spectra are shown in Figure 2. Analyzing the CP-g-OLA-pure filler spectrum shows a peak at around 3200 cm^−1^ assigned to the OH stretching vibration decreased compared to ungrafted CP filler. It is a result of the esterification of the hydroxyl group by LAc, which results in a reduction in OH group concentration. At the same time, the peak around 1735 cm^−1^ assigned to the C=O stretching vibration increased, proving the formation of an ester bond. Y. Luan et al. obtained similar results during grafting cellulose acetate by ROP process [39], the same as A. Goffin et al., who grafted CNW by the ROP process [40]. Additionally, the FTIR spectrum of CP-g-OLA-pure shows new peaks around 1200 and 1090 cm^−1^ assigned to the C-O stretching vibration of OLAs (PLA) backbone [41]. This evidence of chemical structure supports the success of the esterification of hydroxyl groups with LAc and/or OLAs, and incorporating OLAs chains into the macromolecules forming lignocellulosic fillers’ backbones.

#### 3.1.2. Scanning Electron Microscopy of the Fillers

**Figure 3:**
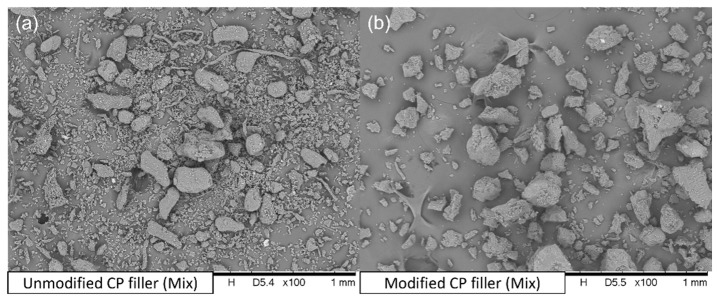
SEM pictures of (**a**) unmodified CP filler; (**b**) modified CP filler.

Figure 3a shows the CP filler after milling. There are significant particle size dispersion and shape differences. It is probably related to the heterogeneous composition of the chokeberry pomace, where you can find stems, fruits, leaves, seeds, etc. Figure 3b presents the CP filler after the modification process. The lower quantity of fibrous particles is likely due to the subsequent crushing of the filler after modification.

#### 3.1.3. Proton Nuclear Magnetic Resonance Analysis

**Figure 4:**
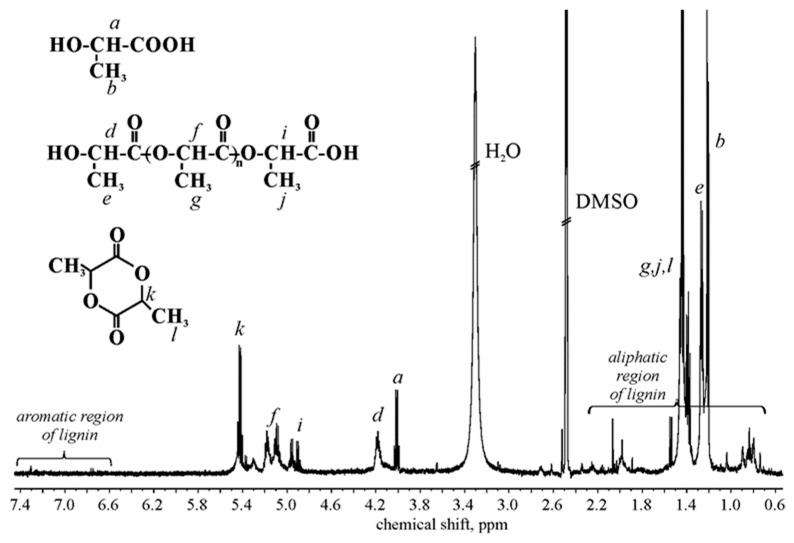
^1^HNMR spectrum of CP-g-OLA.

The determination of the degree of polymerization of OLAs ($\bar{DP}$_OLA_) (grafted and ungrafted), carboxyl group to hydroxyl group ratio ([COOH]/[OH]), the mole fraction of LA (χ_LA_), LAc (χ_LAc_), and OLAs (χ_OLA_) (grafted and ungrafted) was performed using ^1^HNMR analysis (Table 5, Figure 4). $\bar{DP}$_OLA_ value was determined from the equation:

(1) $${\bar{DP}}_{\mathrm{OLA}}=\sum integral\;CH/integral\;CH\left(\mathrm{OH}\;end\right)$$

According to J. Espartero et al., signals from OLAs, LA, and LAc have been assigned in Figure 4 [42]. A [COOH]/[OH] value deviating from 1 suggests that in the spectrum, some protons of OLAs grafted to the lignocellulosic backbone are also seen. The signals of protons present in the backbone of hemicellulose and cellulose are not visible in the spectra due to the insolubility of the backbone in a deuterated solvent, so we are probably dealing with a dispersion that is not recordable under these ^1^H NMR conditions. W. Zhao et al. received signals in the ^1^HNMR spectra in the DMSO-d_6_ of hemicellulose units [43], grafted cellulose (at 80 °C) [44], and lignin [45]. However, it is hard to assign protons to detected signals. In this case, probably aromatic and aliphatic lignin units are shown in shift range 6–8 ppm and 0.5–2.75 ppm, respectively [45]. The presence and content of LA (χ_LA_ = 0.11) is related to used catalyst and process conditions. In the obtained composition, some unreacted LAc is also detected.

#### 3.1.4. Thermal Degradation Analysis of the Fillers

**Figure 5:**
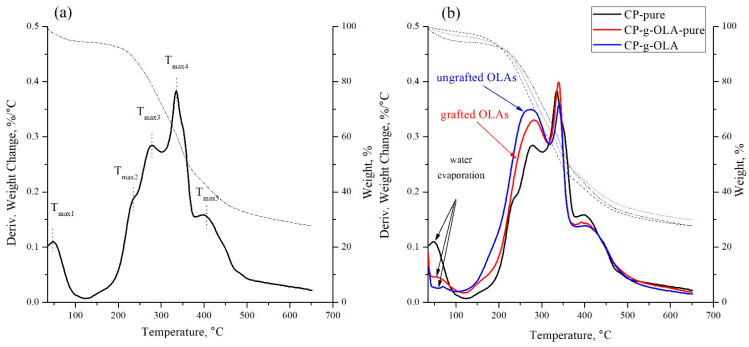
TG and DTG curves of (**a**) CP-pure filler; (**b**) overlaid TG and DTG curves of CP-pure, CP-g-OLA, CP-g-OLA-pure fillers.

TGA analysis was used in order to characterize the fillers (Figure 5a,b).

Based on the TG and DTG curves obtained in TGA analysis, the temperature of 2 and 5% mass loss (T_2%_ and T_5%_, respectively), maximum degradation temperature (T_max_), maximum degradation rate in the third stage of degradation, (V_max3_), and the amount of residue after combustion at 650 °C (R_650_) were determined for all fillers (Table 6).

An analysis of CP-pure filler (Figure 5a) shows multiple peak with three stages of degradation: T_max3_, T_max4_, and T_max5,_ which are assigned to hemicellulose, cellulose/lignin, and lignin degradation, respectively [46,47]. The shoulder with T_max2_ is probably related to tannin degradation [48]. J. Lisperguer et al. obtained a degradation peak for tannin obtained from *Acacia dealbata* at 258 °C [48]. The chemical composition of this specific CP (with no purification) was determined in the previous report by researchers from the Warsaw University of Technology [49]. Table 7 shows the content of cellulose, hemicellulose, lignin, and raw fat in CP filler.

The high content of lignin may positively impact the modification process because of the shielding for hemicellulose and cellulose against humidity and high temperature, as we mentioned at the beginning of the following discussion [27]. The hydroxyl groups which may react with the LAc and OLAs are related to hemicellulose, lignin, and amorphous cellulose. The crystalline cellulose structure is too closely packed (because of hydrogen bonds), which prevents reacting with those hydroxyl groups. Therefore, a high amount of amorphous units (hemicellulose, lignin) content may help to increase the efficiency of the esterification [29,50].

T_2%_ and T_5%_ of CP-pure filler are low (42 ± 4 °C and 71 ± 6 °C, respectively), and it is due to the water evaporation in the weight loss step of maximum temperature T_max1_. After the modification process, T_2%_ value increases significantly, and it can be related to the reduced water absorption by modified, more hydrophobic fillers. The difference in ‘DTG’s curve shape of CP-g-OLA and CP-g-OLA-pure, shown in Figure 5b, in the temperature range around 150–320 °C, is contributed to the grafted and ungrafted OLAs degradation peak superimposed on the peak from tannin and hemicellulose degradation. It is up to a peak with a maximum temperature of approximately 280 °C, and a maximum degradation rate in average range 0.34–0.40%/°C.

Y. Guo et al. also reported an additional T_max_ peak in temperature around 230 °C, corresponding to grafted PLLA chains into the cellulose backbone [51]. N. Burgos et al. synthesized OLAs as a plasticization system for PLA, and also obtained a similar T_max_ degradation of OLAs [36].

In the next step, in the range of 320–400 °C, cellulose and lignin are degraded. The temperature at which the maximum decomposition rate is achieved in this stage is approximately—340 °C.

After the degradation of the CP filler before and after modification at 650 °C, a significant amount of the sample mass remains-approximately 30%. These are probably the tarry residues of lignin pyrolytic degradation.

#### 3.1.5. Water Absorption Analysis

To determine whether the filler has changed its character to be more hydrophobic after the modification, the water absorption was determined. Table 8 shows the water absorption of CP, CP-g-OLA, and CP-g-OLA-pure fillers after 24 h of exposure in equal conditions.

The results show increasing hydrophobicity of the CP filler after the carried out modification both in purified and impurified (with ungrafted OLAs) fillers for 34.5 and 67.3%, respectively. It confirmed that OLAs have a hydrophobic effect on the surface of lignocellulosic fillers by blocking the hydroxyl groups, which was confirmed by FTIR analysis. Additionally, ungrafted OLAs also have a significant impact on improving the hydrophobicity of the lignocellulosic ‘filler’s surface. The ungrafted OLAs are probably physically bound to the surface of the modified filler, and they are shielding hydroxyl groups, which reduces water absorption.

### 3.2. Analysis of Biocomposites

#### 3.2.1. Preparation of Biocomposites

Eight samples of biocomposites comprising 67.09 or 70% of PLA matrix and 30% of various filler fractions or mixtures were prepared via melt mixing in a laboratory extruder at 170 °C for 20 min. A cylindrical profile formed using Ø 1 mm die was collected for further analysis. Detailed information can be found in the Section 2.4.

#### 3.2.2. Scanning Electron Microscopy of the Biocomposites

**Figure 6:**
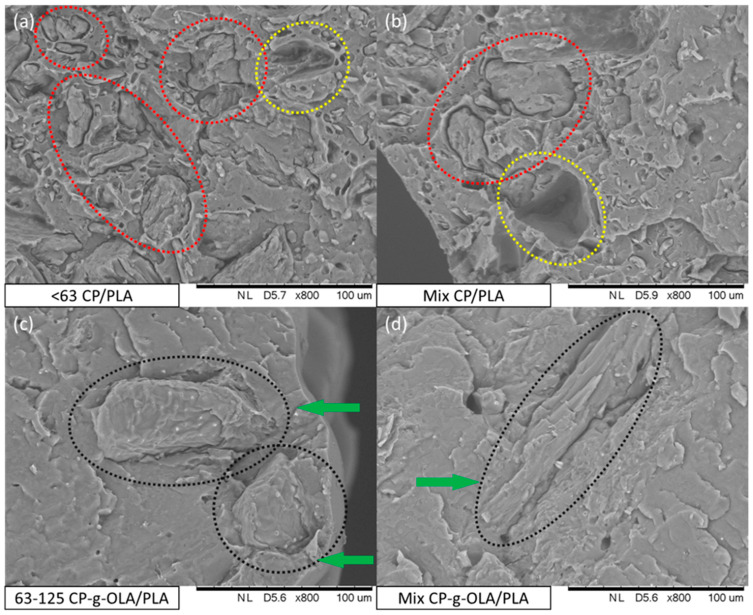
SEM images of (**a**) <63 CP/PLA; (**b**) Mix CP/PLA; (**c**) 63–125 CP-g-OLA/PLA; (**d**) Mix CP-g-OLA/PLA biocomposites.

In order to determine the adhesion between filler particles and the PLA matrix, SEM images of brittle fracture surfaces of biocomposites were obtained (Figure 6).

During the analysis of brittle fracture surfaces of all samples containing particles of unmodified CP filler, it was found that the particles exhibited poor bonding with the PLA matrix. Red-dotted lines in the example SEM images of <63 CP/PLA and Mix CP/PLA samples (Figure 6a,b) indicate areas where CP filler particles can be spotted. Particles are visibly separated from the polymer matrix, and there are no visible indications of good wettability on the particles’ surface by PLA. Moreover, some particles were extracted from the matrix while preparing brittle fractures and left voids (yellow-dotted lines in SEM images in Figure 6a,b). However, surface analysis of brittle fractures of all samples containing CP-g-OLA filler showed that particles exhibited better compatibility and adhesion with the PLA matrix. In the example SEM images of 63–125 CP-g-OLA/PLA and Mix CP-g-OLA/PLA samples (Figure 6c,d), black-dotted lines mark particles, and green arrows point out areas where particles show optimal wettability by PLA matrix. A similar observation was made by Cui et al. during a study involving a modification of cellulose with citric acid [30]. In addition, the lack of <63 µm particles (which are present in the fraction proportions “Mix”; Table 1; Figure 3) on the surface of Mix CP-g-OLA/PLA suggests that these particles were well incorporated into the PLA matrix, proving the effects of modification on better adhesion of CP filler particles with the hydrophobic polymer.

#### 3.2.3. Thermal Degradation Analysis of the Composites

**Figure 7:**
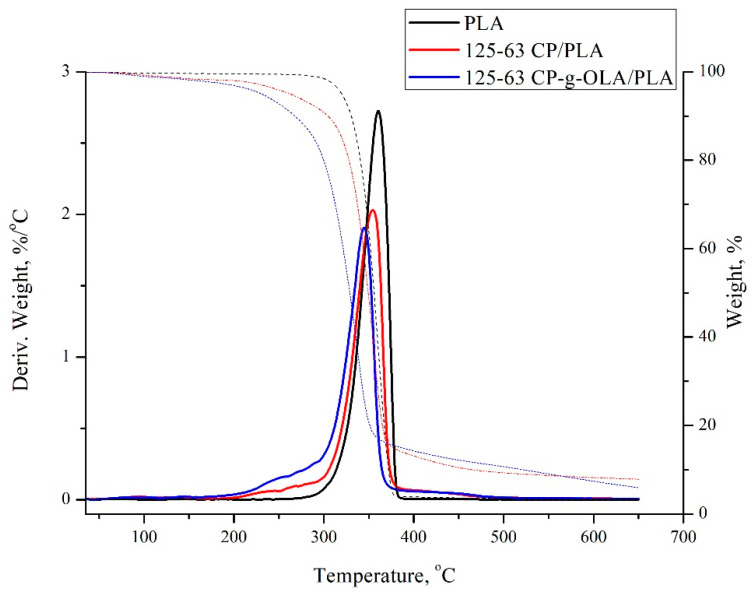
TG and DTG curves of PLA and biocomposites.

Based on the TG and DTG curves obtained in the TGA analysis, the maximum degradation rate (T_max_), the degradation rate (V_max_), and a temperature of 2%, a 5% material loss (T_2%_ and T_5%_) was determined for all biocomposites (Table 9). Representative TG and DTG curves are shown in Figure 7.

TGA analysis showed lower T_2%_, T_5%_, T_max,_ and V_max_ for all biocomposites compared to the unfilled PLA matrix. After adding the lignocellulosic filler, a negative impact on thermal stability is also widely reported [38,52]. Composites are expected to have lower thermal stability due to the presence of lignocellulose filler with lower thermal stability than PLA [52]. The modification and particle size of the lignocellulosic filler do not significantly affect the thermal properties of the analysed biocomposites. Some exceptions are T_2%_ and T_5%_, which are around 20–30 °C lower than composites with an unmodified filler. Similar results were obtained by N. Burgos et al., and are contributed to the low molar mass of OLAs, which makes OLAs more volatile [36]. The wide shoulder in the range of 200–300 °C and the lower V_max_ of biocomposites compared to neat PLA is also related to the content of lignocellulosic filler and OLAs.

#### 3.2.4. Differential Scanning Calorimetry Analysis

**Figure 8:**
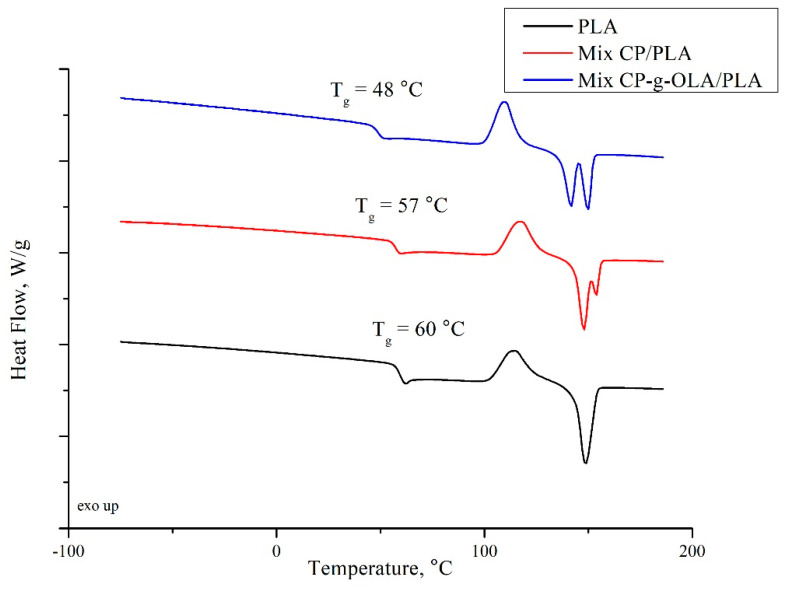
DSC curves of PLA, Mix CP-g-OLA/PLA, and Mix CP/PLA biocomposites.

Glass transition temperature (T_g_), cold crystallization temperature (T_cc_), and melting temperature (T_m_) were determined for all biocomposites using DSC analysis. The crystallinity of the samples (X_c_) was calculated according to the following Equation (2) [2,53,54]:

(2) $$X_{c},\;\%=\frac{\Delta H_{m}-{\Delta H}_{\mathrm{cc}}}{\Delta H_{m\;}^{0}\cdot \omega_{\mathrm{PLA}}}\cdot 100$$

where ${\Delta H}_{\mathrm{cc}}$ is a cold crystallization enthalpy; $\Delta H_{m}$ is a melting enthalpy; $\Delta H_{m\;}^{0}$ is a melting enthalpy of fully crystalline PLA, which is 93.6 J/g [55], and $\omega_{\mathrm{PLA}}$ is a weight fraction of PLA in the biocomposites (Table 4) or in neat PLA. Table 10 and Figure 8 show the results of DSC analysis.

The introduction of lignocellulosic filler into the polymer matrix should increase the T_g_ due to the limitation of the mobility of PLA chains by natural fibers embedded in the matrix [54]. In this case, the T_g_ of neat PLA was found at 60 ± 0 °C; it decreased 2–3 °C after introducing unmodified CP filler. This behavior can be caused by the presence of raw fat in the filler structure (Table 7), which can act as plasticizer of the amorphous phase of PLA. An important finding from this analysis is the significant reduction of T_g_ after the introduction of the modified CP filler into the PLA matrix (average reduction of 8 to 9 °C). R. Avolio et al. have shown that 10% of OLAs may cause the drop of T_g_ by a similar value [35]. In our paper, the total amount of ungrafted OLAs is 2.91% (Table 4). It can be deduced that grafted OLAs also have a plasticizing effect on the PLA matrix, and moreover, the effectiveness of plasticization with OLAs is correlated with their chain length, which in this case is rather low. A. Goffin et al. also obtained a plasticizing effect for pure CNW-g-PLA [40]. To summarize, the decreasing of the T_g_ is connected with the presence of ungrafted and grafted OLAs. It confirms the plasticizing effect of the chain of OLAs in this composition. Additionally, DSC curves showed only one T_g_ in the analyzed temperature range, which shows no macroscopic phase separation and good compatibility between the PLA and OLAs in analyzed biocomposites [35].

The addition of unmodified CP filler increase T_cc_ which can correspond to the limitation of PLA’s chains mobility. Introduction OLAs to the PLA matrix generally caused a slight decrease in T_m1_. This phenomenon is also related to the plasticizing effect of OLAs and increasing PLA chain mobility [35]. Except for the unfilled PLA matrix, samples were characterized by a double melting peak (T_m1_ and T_m2_). This effect is widely known in the literature as α’ phase melting (T_m1_), and its recrystallisation to α and re-melting at higher temperature (T_m2_) [56]. Lower T_m2_ is characterized in all grafted CP fillers, which could contribute to higher nucleation of crystallization by CP-g-OLA because of the higher mobility of PLA chain. It is clearly shown that the introduction of the filler affects the process of nucleation, thus increasing the X_c_ polymer matrix [11]. According to the literature, OLAs also have an increasing impact on the degree of crystallinity [35].

#### 3.2.5. Mechanical Properties

Table 11 shows Young’s modulus and tensile strength of unmodified and modified biocomposites.

**Figure 9:**
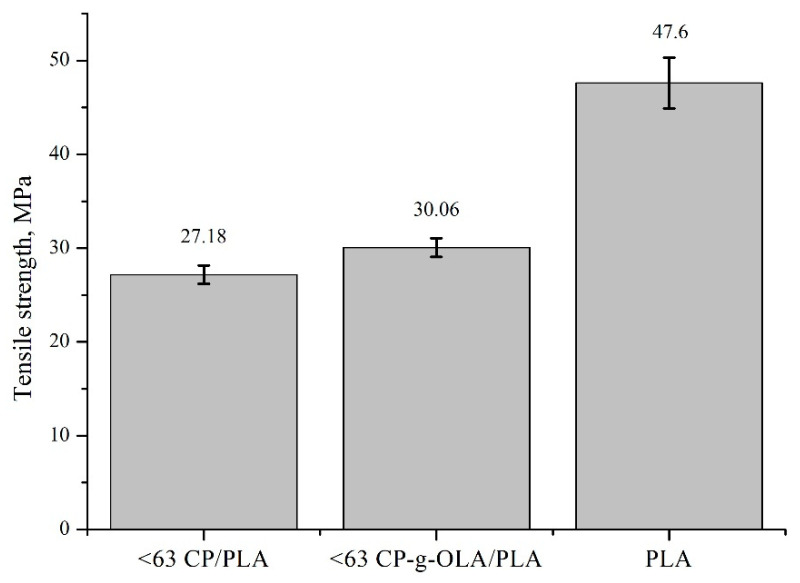
Tensile strength of neat PLA, <63 CP/PLA and <63 CP-g-OLA/PLA.

All of the biocomposites are characterized by a lower tensile strength than neat PLA. It is quite an obvious observation, given the degree of filling (30%) of the composite and the poor adhesion of the unmodified filler to the matrix. A. Dufresne et al. also observed this effect after adding lignocellulosic flour to the PHBV [6]. An upward trend is clearly visible in the case of the tensile strength of composites with modified fillers compared to unmodified fillers. The increase is slight, and often on the verge of standard deviation in most cases. It is important to notice that generally, in this case, plasticizers and OLAs reduce tensile strength [35,36,57]. In this composition, good adhesion and wettability (which is proven by SEM analysis, Figure 6c,d) may compensate for the reduction in tensile strength caused by the addition of the plasticizer. The highest increase in tensile strength of the biocomposites with a modified filler (comparing with biocomposites with unmodified filler) is shown in the biocomposites with a filler in the size fraction <63 µm and is around 10%. This result can be justified as a greater development of the surface area, which results in a higher number of hydroxyl groups on the surface, able to react with OLAs and LAc [58]. Figure 9 presents this result.

## 4. Conclusions

In this paper, CP, which is a food-based residue, was modified using in situ formed OLAs to increase the adhesion between the filler particles and PLA matrix. The esterification reaction results in the mixture of ungrafted and grafted OLAs to CP particles. FTIR spectra confirmed the success of the grafting process. SEM images confirm better adhesion between PLA matrix and modified filler than with unmodified CP filler. DSC results showed the plasticization effect of ungrafted and grafted OLAs, with decreasing T_g_ on average by 9 °C. The water absorption of a modified CP filler is lower than an unmodified filler by 67.3%, and confirms participation in blocking hydroxyl groups also by ungrafted OLAs. Thanks to a better connection of modified CP filler to PLA matrix, the mechanical properties of biocomposites were less deteriorated than in the case of an unmodified natural filler. Mechanical and thermal tests have shown that the use of a mixture of fractions of a different size (Mix 1) than the original (Mix) does not have a significant effect on the properties of biocomposites. However, it seems that the smallest particles (less than 63 µm) have the best impact on the properties of obtained biocomposites. The research results suggest that the produced biocomposites with a natural plasticizer will have potential applications in the production of food packaging.

## Figures and Tables

**Table 1:** Fraction proportions of milled CP filler (Mix).

Type of Filler	Fraction, µm	Fraction Content, %	Name of Fraction Proportion
CP	250–125	32.7	Mix
	125–63	49.1	
	<63	18.2	

**Table 2:** Modification details.

CP Filler, g	Water Content in CP Filler, %	DL-Lac ^1^, g	SnCl_2_·2H_2_O ^1^, mg	Total Water, g
180	4.0	180	39.4	630

^1^ With respect to the 100% reactants.

**Table 3:** Fraction proportions of grinded CP filler after the modification (Mix 1).

Type of CP Filler	Fraction, µm	Fraction Content, %	Type of Mix
CP-g-OLA	250–125	43.5	Mix 1
	125–63	33.4	
	<63	23.1	

**Table 6:** TGA results of the fillers.

Sample	The Characteristic Temperature of Thermal Decomposition, °C							V_max3_, %/°C	R_650_, %
	T_2%_	T_5%_	T_max1_	T_max2_	T_max3_	T_max4_	T_max5_		
CP-pure	42 ± 4	75 ± 8	52 ± 2	237 ± 3	278 ± 0	335 ± 1	398 ± 1	0.29 ± 0.01	30 ± 1
CP-g-OLA	106 ± 4	169 ± 10	76 ± 7	-	262 ± 11	341 ± 1	404 ± 3	0.40 ± 0.05	25 ± 3
CP-g-OLA-pure	71 ± 6	172 ± 3	67 ± 10	-	283 ± 1	338 ± 1	394 ± 2	0.34 ± 0.01	30 ± 1

**Table 7:** Chemical composition of CP filler [49].

Filler	Raw Fat, %	Cellulose, %	Hemicellulose, %	Lignin, %
CP	7.3	20.6	21.7	58.0

**Table 8:** Water absorption results.

Sample	Water Content, %
CP	5.5
CP-g-OLA-pure	3.6
CP-g-OLA	1.8

**Table 9:** TGA results of PLA and biocomposites.

Sample	T_2%_, °C	T_5%_, °C	T_max_/V_max_, °C/%/°C
PLA	305 ± 1	320 ± 1	360 ± 1/2.78 ± 0.05
<63 CP/PLA	184 ± 1	251 ± 2	350 ± 3/2.15 ± 0.13
63–125 CP/PLA	201 ± 1	259 ± 6	347 ± 12/1.93 ± 0.19
Mix CP/PLA	194 ± 4	254 ± 5	321 ± 5/1.66 ± 0.32
Mix1 CP/PLA	198 ± 2	254 ± 12	350 ± 6/2.01 ± 0.11
<63 CP-g-OLA/PLA	173 ± 1	231 ± 1	346 ± 2/2.08 ± 0.02
63–125 CP-g-OLA/PLA	178 ± 0	234 ± 3	343 ± 4/1.79 ± 0.20
Mix CP-g-OLA/PLA	174 ± 2	231 ± 1	346 ± 2/1.96 ± 0.04
Mix1 CP-g-OLA/PLA	181 ± 4	230 ± 1	343 ± 1/1.69 ± 0.34

**Table 10:** Results of DSC analysis.

Sample	T_g_, °C	T_cc_, °C	T_m1_/T_m2_, °C	X_c_, %
PLA	60 ± 0	115 ± 0	149 ± 0/-	1.8 ± 0.3
<63 CP/PLA	58 ± 0	121 ± 1	149 ± 0/154 ± 1	3.4 ± 0.5
63–125 CP/PLA	58 ± 0	121 ± 2	149 ± 1/154 ± 1	4.0 ± 1.2
Mix CP/PLA	57 ± 0	118 ± 1	148 ± 1/154 ± 1	3.3 ± 1.1
Mix1 CP/PLA	58 ± 0	120 ± 1	149 ± 0/153 ± 1	4.4 ± 1.1
<63 CP-g-OLA/PLA	52 ± 1	117 ± 4	145 ± 2/151 ± 1	4.2 ± 0.9
63–125 CP-g-OLA/PLA	51 ± 2	118 ± 6	145 ± 3/151 ± 1	2.5 ± 0.3
Mix CP-g-OLA/PLA	51 ± 3	118 ± 7	145 ± 3/151 ± 1	5.1 ± 0.3
Mix1 CP-g-OLA/PLA	52 ± 1	121 ± 4	146 ± 1/152 ± 1	4.4 ± 0.4

**Table 11:** Mechanical properties of biocomposites.

Sample	Tensile Strength, MPa
PLA	47.6 ± 2.7
<63 CP/PLA	27.18 ± 0.99
63–125 CP/PLA	26.19 ± 1.08
Mix CP/PLA	26.37 ± 1.08
Mix1 CP/PLA	25.92 ± 0.90
<63 CP-g-OLA/PLA	30.06 ± 0.99
63–125 CP-g-OLA/PLA	27.63 ± 1.08
Mix CP-g-OLA/PLA	27.81 ± 1.17
Mix1 CP-g-OLA/PLA	27.63 ± 1.62

## Data Availability

The data presented in this study are available on request from the corresponding author.
